# Comparison Between Helpful and Missing Resources Identified by Patients with End-Stage Liver Disease and Their Caregivers: A Content Analysis †

**DOI:** 10.3390/nursrep16030095

**Published:** 2026-03-09

**Authors:** Susan J. Rosenkranz, Shirin O. Hiatt, Amy Leatherwood, Michael F. Chang, Lissi Hansen

**Affiliations:** 1 School of Nursing, Oregon Health & Science University, 3455 S.W. U.S. Veterans Hospital Rd., Portland, OR 97239, USA; hiatts@ohsu.edu (S.O.H.); leatherw@ohsu.edu (A.L.); hansenli@ohsu.edu (L.H.); 2 Gastroenterology & Hepatology, VA Portland Health Care System, 3710 S.W. U.S. Veterans Hospital Rd., Portland, OR 97239, USA; michael.chang2@va.gov

**Keywords:** end-stage liver disease, liver failure, palliative care, health resources, social support, patient unmet needs, caregiver unmet needs

## Abstract

Patients with end-stage liver disease (ESLD) and their caregivers experience extensive physical, psychological, and social burdens and needs for resources. However, empirical evidence on patients’ and caregivers’ specific reported use of resources to help manage ESLD is lacking. Understanding the type and helpfulness of resources used could strengthen clinical care to address individual needs for resources. **Aim:** To examine and compare resources patients and caregivers identified as being most helpful in managing ESLD in relation to resources they felt would be helpful. **Methods:** Patients with ESLD and their caregivers responded in writing to two open-ended questions as part of a survey: (1) What resources have you found most helpful in dealing with patient’s liver disease? and (2) What resources would be helpful in dealing with patient’s liver disease? Conventional content analysis was used to identify resource categories. **Results:** A total of 192 patients and 198 caregivers completed surveys. We identified two major resource domains—medical and non-medical—and five categories within each. Analysis revealed participant group- and disease severity-based differences in helpful resources. **Conclusions:** Proactively engaging patients and caregivers early in the course of illness to identify relevant resources that might facilitate ability to manage ESLD. An interprofessional care approach would facilitate efforts supporting financial, social, spiritual, emotional, and mental health needs. Future longitudinal research of unique resource needs along the disease trajectory may help to develop effective interventions.

## 1. Introduction

In the U.S., an estimated 4.5 million individuals have chronic liver disease (CLD), and 633,000 have cirrhosis, a scarring of the liver caused by various etiologies [1]. Cirrhosis exists in two forms: compensated and decompensated. Compensated cirrhosis is a stage of liver disease where the liver is severely scarred but able to compensate for the damage and may cause mild or undetectable symptoms. Decompensated cirrhosis is a more advanced form and patients often experience complex, debilitating, and interrelated symptoms that they and their informal caregivers manage daily outside of clinical settings [2,3]. The cirrhosis at this point is irreversible and the median survival is 2 years [4,5]. Both patients living with decompensated cirrhosis and their informal caregivers (referred to as “caregivers” hereafter) face extensive physical, psychological, and social burdens [3]. As patients’ health deteriorates to end-stage liver disease (ESLD), need for healthcare services, resources, and support increases for both patients and their caregivers. Investigators have concluded that healthcare services available to patients and their caregivers are frequently incompatible with their unique needs related to liver disease and end of life care [6,7]. Research on healthcare services have focused on hospitalizations, intensive treatments, and associated costs by disease severity, whereas there is a lack of empirical evidence on the resources patients with ESLD and their caregivers use and need to manage and live with ESLD [8,9,10]. Resources can be defined as materials, staff, and other assets that can be drawn on by a person to function effectively [11].

There is evidence that healthcare professionals (HCPs) may not address the needs of patients with CLD or provide the services or resources that they should have. A mixed-methods systematic review of patients with CLD found patients wanted to know more about liver disease, to understand it, and to have information that would help them manage it better [12]. Patients have reported that the information they receive is too medicalized [13,14] and that their unmet needs related to information and support from HCPs severely affect their quality of life [12].

The significant burden and unmet needs of caregivers of patients with advanced liver disease and liver transplant candidates have been reported [2,3,15]. Family caregivers have reported feeling unprepared, insufficient communication from HCPs, and need for information about the disease, symptoms, treatments, caregiving, and what to expect as the disease progresses, as well as need for financial and mental health supports [3,15]. Successful strategies to mitigate these unmet needs of caregivers and the burden they experience that have a negative impact on their quality of life are scarce [3]. Baja et al. [16] showed that a mindfulness-based stress reduction intervention reduced caregiver burden and depression in both caregivers and patients with cirrhosis. Other interventions have been suggested that test psychological support, coping strategies, and educational materials on the health and well-being of caregivers of patients with cirrhosis [2]. Research is lacking on the actual resources caregivers find helpful in caring for persons with ESLD and less attention is given to understanding differences in helpful resources between patients with ESLD and their caregivers. By learning more about helpful resources used by both patients and caregivers as well as identifying resources they feel *would be* helpful, clinical care could be strengthened to address their similar and uniquely different needs related to these resources. Therefore, the purpose of this qualitative analysis was to examine and compare resources patients and caregivers identified as being most helpful in managing ESLD in relation to resources they felt would be helpful.

## 2. Materials and Methods

### 2.1. Study Design

This was a content analysis of patients’ with ESLD and caregivers’ written responses to two open-ended questions as part of a survey collected at baseline for a larger longitudinal study in the U.S. Pacific Northwest [17]. The larger study design and data collection has been described in detail elsewhere [17]. The study was approved by a joint institutional review board (IRB#9516) [17]. Prior to participation in the study, all individual patients and caregivers provided written informed consent. Baseline data including the two open-ended questions for this content analysis were collected from May 2016 through December 2018.

### 2.2. Sample

Outpatient hepatology clinics within two healthcare organizations were the source of recruitment of patients with ESLD and their caregivers. Healthcare providers in the clinics participated in screening patients for study eligibility when patients came for their liver clinic appointments. Patient eligibility criteria included ≥21 years of age and a score of ≥15 on Model for End-Stage Liver Disease-Sodium (MELD-Na) [18]. The MELD-Na score was available during data collection but the more recent implemented MELD 3.0 was used for the analysis [19]. The MELD, a numerical scoring system, is used to assess liver disease severity and is an independent predictor of mortality in patients with liver cirrhosis over a 90-day period [20]. The score is calculated based on sex and a blood sample including bilirubin, sodium, international normalized ratio (INR), creatinine, and albumin. It ranges from 6 to 40, with higher scores reflecting more severe liver disease (dysfunction) and higher mortality rate. The MELD is used to determine patient eligibility for liver transplantation. MELD 3.0 has shown to better predict mortality compared to MELD-Na and particularly among females. All laboratory values to calculate the MELD 3.0 for the sample were the ones collected closest to patients’ clinic appointment or the day of their appointment and available before the time of enrollment. MELD 3.0 score calculations were completed by healthcare providers and did not include missing values.

Immediately after the appointment, the HCP provided patients identified as study eligible and their caregiver the opportunity to meet with a study team member at the clinic to learn more about the study and informed them that study participation would not affect their care. Patients and caregivers that expressed interest then met with a study team member who described the study, determined caregiver eligibility, and invited study participation. Caregiver eligibility criteria included individuals identified by patients as the primary person age ≥ 18 who provided unpaid help and assistance (e.g., a spouse, significant other, sibling, adult child, adult grandchild, or friend). Patients and caregivers were enrolled in the study consecutively based on the day and time they came for the patient’s appointment.

### 2.3. Data Collection

Once enrolled in the study, a study team member gave patients and caregivers each a survey packet to complete and return in the self-addressed stamped envelope at their convenience. Patients and caregivers were provided with the option to receive the survey packet in the mail. The survey packet included a self-administered written questionnaire that included 11 demographic questions and the following two open-ended questions:

1. What resources have you found *most helpful* in dealing with your (the care recipient’s) liver disease?
2. What resources *would be helpful* in dealing with your (the care recipient’s) liver disease?

Participants could write as many resources as desired in response to each question.

A study team member collected patient stage of disease, primary disease etiology, MELD-Na scores and evidence of hepatic encephalopathy and ascites from patient medical records when available.

### 2.4. Data Analysis

Participant written responses to open-ended questions were transcribed verbatim and verified by two members of the research team. Data were uploaded to and managed with NVivo 11 qualitative software [21]. The responses were analyzed by a coding team consisting of four research team members (SJR, SOH, AL, LH) using qualitative conventional content analysis as an inductive and structured approach to identify resource categories from the perspectives of patients and caregivers and to summarize findings [22,23,24]. This approach, as described by Hsieh and Shannon [22], is a form of descriptive qualitative analysis to manage data from open-ended questions and to interpret meaning from the content of text data. In this qualitative approach, coding categories were derived inductively directly from patient and caregiver written responses to the open-ended questions. We initially identified 73 codes that were subsequently clustered into categories based on similar or overlapping meaning and relationship as a resource. This methodology offers a straightforward and less interpretive presentation of everyday language [25]. After all data were collected, the coding team individually read through all participant responses to obtain a sense of the entire data set and re-read responses word by word to identify initial codes that capture resources as described by participants. Then, all four coders met to compare coding and develop a codebook based on the agreed-upon codes. Next, each team member independently applied the codebook to the entire data set; pairs of coders met weekly to compare interpretations and applications of codes and resolve coding differences through discussion. Once all data were coded, the entire coding team met to group codes similar in concept to describe overarching resource categories. For example, the codes “online research” and “print material” were grouped as *liver disease knowledge* resource category. The coding team used various data sets produced throughout analysis (participant responses, codes, categories, meeting minutes, memos) to secure data triangulation. Individual responses that included at least one word (e.g., family) were included in the analysis. The number of words in an individual response did not influence how codes were applied. The absence of a written response to either question 1 or 2 was considered a missing response and excluded from final analysis for that specific question; participants were retained in analyses for questions they answered. In addition, qualitative coding was complemented by a count of the coded categories to summarize the data, interpret patterns highlighted in the process, and compare resources by question, participant group (patient vs. caregiver), and disease severity [25,26,27]. For our analysis, this “quantitative” (counting/percentages) analysis was a subset of the qualitative content analysis process [26,27]. Throughout the process, the entire coding team met bi-weekly to discuss category development and resolve coding discrepancies to reach group consensus to enhance the trustworthiness of the analysis [28]. To ensure credibility and dependability, the team maintained an audit trail through documented decisions around codebook and category development, meeting minutes and memos [28]. A frequency count of each resource category was developed based on the number of participants whose responses were coded in a particular category and used to identify the most commonly recurring categories in response to each question across and within participant groups [26,27]. Standard descriptive statistics were used to describe the demographic characteristics of respondents. The category financial status included four response options: (1) Can’t make ends meet; (2) Have just enough, no more; (3) Have enough, with a little extra sometimes; (4) Always have money left over.

## 3. Results

A total of 192 patients and 198 informal caregivers completed surveys; 182 patients and 178 caregivers responded to question 1, and 156 patients and 151 caregivers responded to question 2. Most patients belonged to Child classes B and C. The Child–Pugh score measures the severity of CLD and predict prognosis, from well-compensated (Child class A) to decompensated (Child class C) [29]. Table 1 describes both patients’ and their caregivers’ demographics.

The number of resources each participant reported in response to each question ranged from one to eight. In response to question 1, patients and caregivers listed a total of 423 and 407 resources respectively that they found *most helpful*. In response to question 2, patients and caregivers reported a total of 244 and 252 resources respectively that they felt *would be helpful*.

### Overview of Responses

Patients and caregivers described a wide range of most helpful used and desired resources. To obtain a sense of the most helpful resources and needed resources, we counted the number of participants whose responses included a particular resource category code. We focused on the 10 most frequently reported categories across both patients and caregivers. Table 2 and Table 3 present the top 10 resource categories that occurred most frequently across all responses to each question; the percentage of combined patient and caregiver responses are indicated. Resource categories were grouped within two major domains: (1) non-medical and (2) medical. The non-medical domain addresses resources located outside of the health care system and patient–HCP interactions and includes five primary categories: *social support*, *religious belief system/spirituality*, *support groups*, *financial*, and *resource awareness* (Table 2).

The medical domain describes resources situated within the medical system and patient–HCP interactions and includes five main categories: *HCPs/health care system*, *information from HCPs*, *liver disease knowledge*, *access to medications/procedures*, and *mental health services* (Table 3).

**Figure 1:**
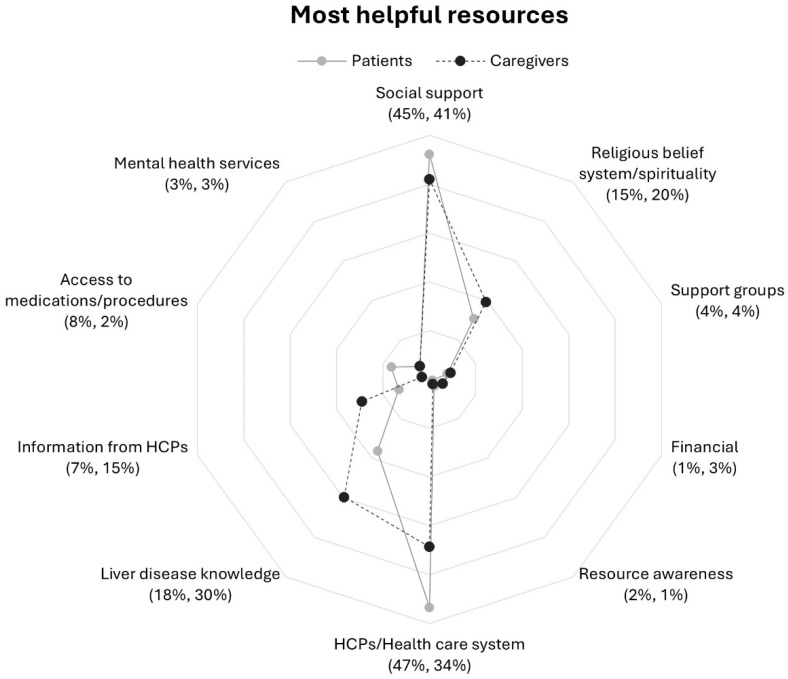
Proportions of patients and caregivers reporting resources in response to question 1 by participant group: *“What resources are most helpful in dealing with your (the care recipient’s) liver disease?”*. Note. Patients’ (*n* = 182) response rate percentage is listed first followed caregivers (*n* = 178) response rate percentage. Abbreviation: HCPs, health care professionals.

**Figure 2:**
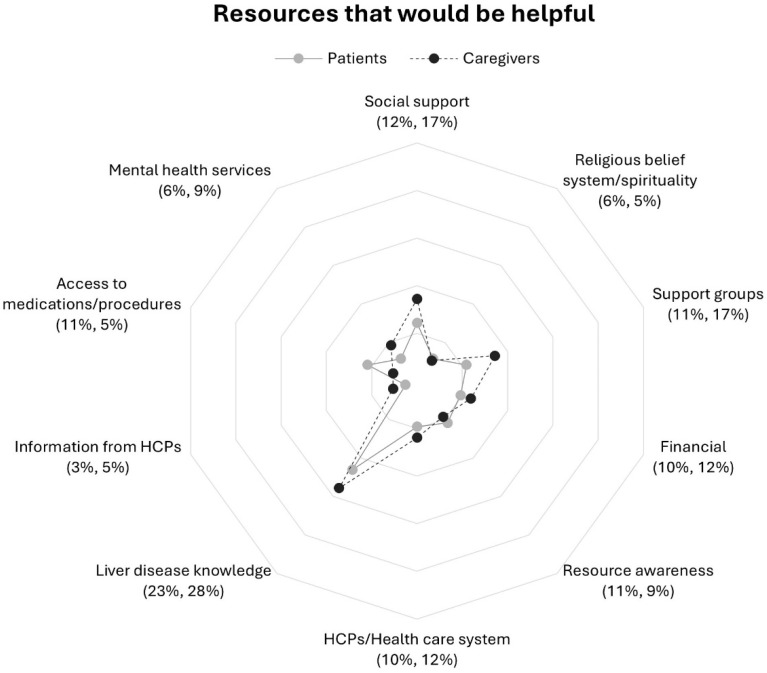
Proportions of patients and caregivers reporting resources in response to question 2 by participant group: *“What resources would be helpful in dealing with your (the care recipient’s) liver disease?”*. Note. Patients (*n* = 156) response rate percentage is listed first followed by caregivers’ (*n* = 151) response rate percentage. Abbreviation: HCPs, health care professionals.

There were increases between question 1 and 2 in response rate percentage across all categories with frequencies of 5% and lower at question 1 (Table 2 and Table 3). Rates of response to each question from patients and caregivers demonstrated similarities within most categories to questions 1 and 2 but distinct contrasts within the medical domain in question 1 (Figure 1 and Figure 2).

A descriptive summary of salient resource categories identified through content analysis of patient and caregiver responses to each question are presented below and ranked from most to least frequently occurring within non-medical and medical domains. Differences in the occurrence of resource categories by patient’s Child–Pugh score (Child class) are also highlighted [29].

**Question 1: Most helpful Resources Used**

Responding to question 1, both patients and caregivers were most likely to mention resources described by the category social support from the non-medical domain, followed by two medical domain resources HCPs/health care system and liver disease knowledge (Table 2 and Table 3). The next most frequently reported categories were religious belief system/spirituality and information from HCPs. Resource categories with a combined response rate of 5% or less included access to medications/procedures, support groups, mental health services, financial, and resource awareness. A comparison of patient and caregiver response frequencies is shown in Figure 1.

**Non-medical domain**

**Social support.** Both patient and caregiver groups reported support from family, friends, and others as a helpful resource at similar rates. (Figure 1). Talking with someone who could relate to the experience of having liver disease or caregiving was helpful for both patients and caregivers. Caregivers included practical (instrumental) assistance with patient care.

**Religious belief system/Spirituality.** Overall, caregivers reported this category at a slightly higher rate than patients (Figure 1). Both patients and caregivers often mentioned church, prayer, and “talking with God” in responses. Patients in general turned to their religious belief system or spirituality for guidance and to “stay focused spiritually.” Caregivers wrote of spirituality, meditation practice, and found comfort in the Bible and their faith as a form of coping.

**Medical domain**

**Health care professionals/Health care system.** Patients reported this category at a higher rate than caregivers (Figure 1). Overall, respondents highlighted perceived quality of care received from medical institutions and the medical teams involved in their care as helpful resources. Caregivers were more likely than patients to report on the quality of interactions with HCPs (i.e., “personable,” “caring,” and “understanding”).

**Liver disease knowledge.** Patients reported this resource at a much lower rate as compared to caregivers (Figure 1) and often identified their caregiver as the person seeking information. Caregivers found it helpful to educate themselves not only about liver disease but also liver transplantation and the evaluation process, caregiving, and what questions to ask HCPs during office visits. Respondents utilized online sources such as “WebMD online” and print materials to learn about liver disease and to augment a perceived lack of information provided by HCPs, as one caregiver noted: “Information looked up on Google; Very limited information from DR.”

**Information from HCPs.** Caregivers reported this category at a higher rate than patients (Figure 1). Respondents described communicating with their HCPs to obtain information about their care, liver disease in general, and interpreting labs and symptoms. Receiving information from HCPs early in the diagnosis was helpful particularly for caregivers. Patients and caregivers alike appreciated receiving direct and honest information about the patient’s condition from their HCP and gathering information directly from HCPs during office visits.

**Child–Pugh score-based differences**

**Figure 3:**
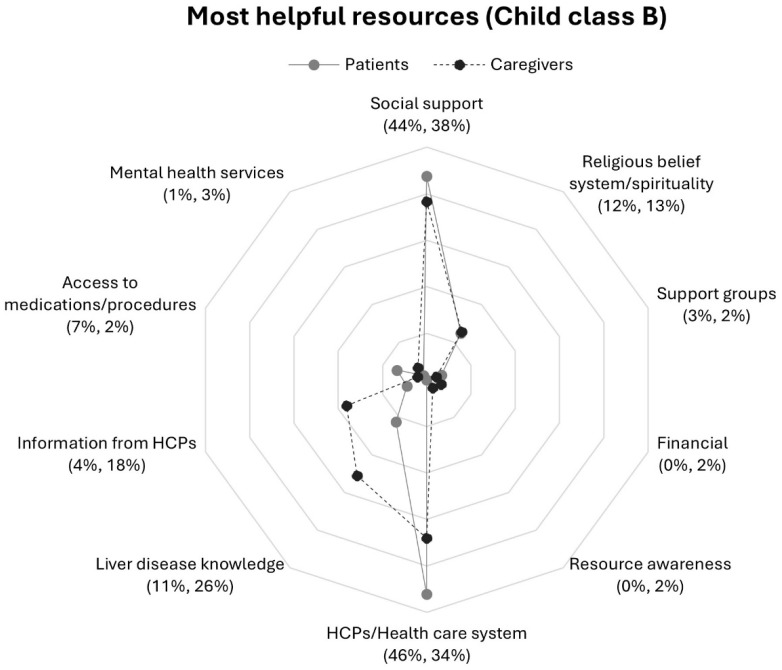
Proportions of patients and caregivers reporting resources in response to question 1 by participant Child class B: *“What resources are most helpful in dealing with your (the care recipient’s) liver disease?”*. Note. Patients’ (*n* = 89) response rate percentage is listed first followed caregivers (*n* = 94) response rate percentage.

**Figure 4:**
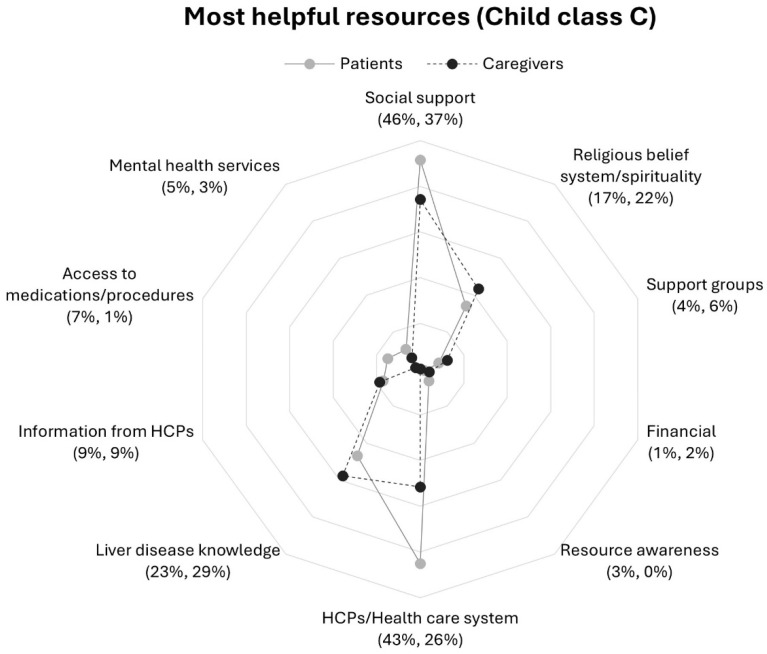
Proportions of patients and caregivers reporting resources in response to question 1 by participant Child class C: *“What resources are most helpful in dealing with your (the care recipient’s) liver disease?”*. Note. Patients (*n* = 94) response rate percentage is listed first followed by caregivers’ (*n* = 97) response rate percentage. Abbreviation: HCPs, health care professionals.

A comparison of Child–Pugh scores revealed differences in category frequencies (Figure 3 and Figure 4). Patients in Child class C as compared to class B were more than five and two times as likely to report mental health services and liver disease knowledge respectively. Among caregivers, those in Child class B reported information from HCPs twice as frequently as compared to those in Child class C; response frequencies for social support were three times higher in Class C than Class B.

Contrasts in response rates were also observed between patients and caregivers within a Child–Pugh class. Patients in Child class C were more likely to mention mental health services than caregivers in their class (Figure 4). Notably, patients and caregivers within Child class C mentioned information from HCPs at the same rate, a distinct contrast to the differences between patient and caregiver frequencies within Child class B and as compared to patients and caregivers overall (Figure 1).

**Question 2: Resources That Would be Helpful**

Patients and caregivers combined most frequently reported resources in the liver disease education category within the medical domain (Table 3), followed by social support in the non-medical domain (Table 2). Notably increases in combined patient–caregiver response rate frequences in response to question 2 as compared to question 1 were observed in non-medical domain resources—support groups, financial, and resource awareness (Table 2). In contrast, HCP/health care services and information from HCPs in the medical domain were less likely to be mentioned in response to question 2 as compared to question 1. A comparison of patient and caregiver response frequencies is shown in Figure 2.

**Non-medical domain**

**Social support.** Overall, patients emphasized wanting support from family, but caregivers were more likely to mention non-family members (i.e., friends, co-workers, etc.) (Figure 2). Patients and caregivers alike expressed a desire for social interactions with other people that could relate to their experiences of living with liver disease or caregiving. Caregivers wanted help with practical activities and expressed a desire for family members to share in caregiving.

**Support groups.** Caregivers as compared to patients mentioned resources in this category at a higher rate (Figure 2). In general, patients desired patient-centered support groups to improve understanding of symptoms and for the shared experience of having liver disease, with a preference for groups specific to disease etiology or transplant status. Caregivers wanted opportunities to interact with other caregivers to share experiences and support groups that were “local” and “in community.”

**Financial.** Caregivers more than patients described resources in this category including access to or assistance with medical expenses, food, housing, or transportation to appointments (Figure 2). For patients, finances were a source of stress and mentioned in context of job security and being unemployed. Caregivers also mentioned income reduction, but more often emphasized access to health insurance and help with medical and non-medical expenses (e.g., food, housing, home improvements, and transportation).

**Resource awareness.** Patients and caregivers indicated a lack of awareness of and uncertainty about helpful resources at similar rates (Figure 2). Patients described the potentially overwhelming experience of managing liver disease at home. Similarly, caregivers were “not sure” about what additional resources they may need or lacked awareness of what might be available to them.

**Medical domain**

**Liver disease education.** Patients wanted to learn more about their specific diagnosis and help with interpreting test results but were less likely than caregivers to report this category (Figure 2). Caregivers were concerned with information about surgeries, hepatic encephalopathy “and its effects,” caregiving, and knowing what to expect as the disease progresses. Caregivers suggested a family member-centered class about liver disease and wanted access to liver disease education early in the diagnosis process.

**HCPs/health care system.** Patients and caregivers responded at similar rates within this category (Figure 2). Patients wanted “timely,” frequent, and “regular support” from HCPs and indicated a preference for specialists. In contrast, caregivers desired more communication from HCPs “following appointments” and improved communication between care teams. Access to local HCPs and “closer facilities” was also mentioned by caregivers.

**Access to medications/procedures.** Patients were more than twice as likely as caregivers to report this category (Figure 2). Across both patient and caregiver responses, focus shifted from addressing symptoms and towards curative treatment (i.e., liver transplantation). Patients highlighted the requirements for receiving a transplant, but caregivers expressed a sense of urgency for liver transplantation and wanted a better understanding of treatment decisions from HCPs.

**Mental health services.** Caregivers were more likely than patients to mention mental health services (Figure 2). A need for improved access to mental health support on a regular basis, but also during and after hospitalization was evident across responses. Some patients desired more frequent and one-on-one counseling. Caregivers mentioned one-on-one counseling for themselves but also for the patient.

**Child–Pugh score-based differences**

**Figure 5:**
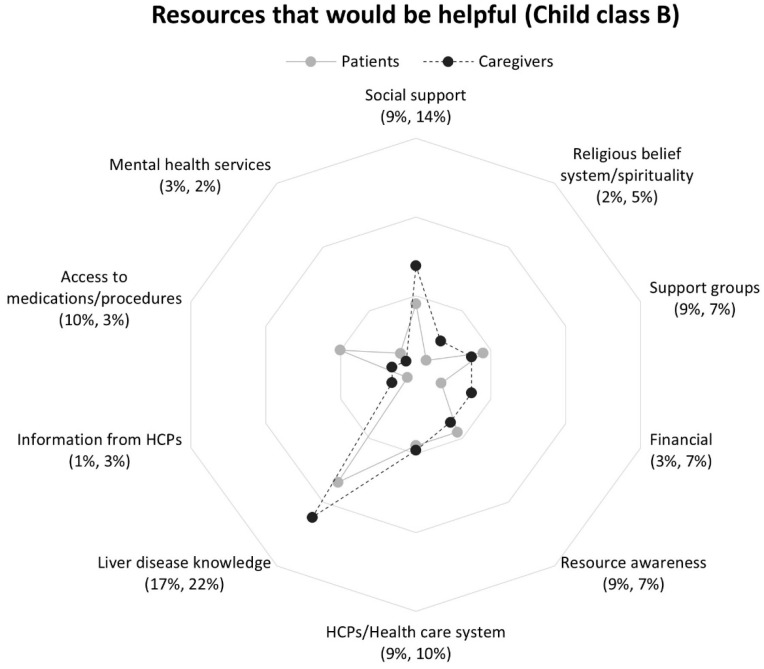
Proportions of patients and caregivers reporting resources in response to question 2 by patient Child class B: *“What resources would be helpful in dealing with your (the care recipient’s) liver disease?”*. Note. Patients’ (*n* = 89) response rate percentage is listed first followed caregivers (*n* = 94) response rate percentage. Abbreviation: HCPs, health care professionals.

**Figure 6:**
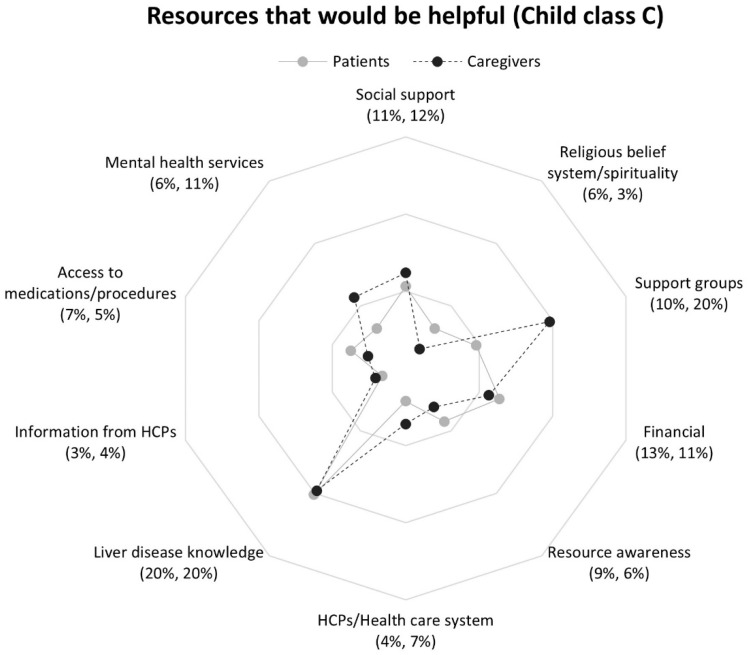
Proportions of patients and caregivers reporting resources in response to question 2 by patient Child class C: *“What resources would be helpful in dealing with your (the care recipient’s) liver disease?”*. Note. Patients (*n* = 94) response rate percentage is listed first followed by caregivers’ (*n* = 97) response rate percentage. Abbreviation: HCPs, health care professionals.

Respondents in Child classes B and C most frequently reported liver disease knowledge, although caregivers in Child class C also mentioned support groups at the same frequency (Figure 5 and Figure 6). Importantly, resource awareness (lack of) was reported by all respondents at nearly the same rate, regardless of Child class. Child–Pugh score-based differences in response frequencies were observed across non-medical and medical domains. Respondents in Child class B were more likely than Child class C to report HCPs/health care system resources. In contrast, respondents in Child class C more often mentioned financial, support groups, and mental health services than Child class B respondents.

Child-Pugh score-based differences were observed within Child classes between patient and caregiver responses. Within Child class B, patients were three times more likely than caregivers to mention access to medications/procedures (Figure 5), but response rates between patients and caregivers were more closely aligned within Class C (Figure 6). Among respondents in Class C, caregivers reported support groups twice as frequently, and nearly two times more often mentioned a desire for mental health services compared to patients, Figure 6.

## 4. Discussion

This study examined and compared resources patients living with ESLD and their caregivers identified as being most helpful in managing ESLD in relation to resources they felt would be helpful. Through content analysis of all responses to two open-ended questions, we identified two domains of resources across the 10 most frequently occurring categories among respondents: (1) medical and (2) non-medical. These domains highlight that patients and caregivers use and want access to resources from across both domains. However, our analysis shows that the types of resources differ between those used and those that would be helpful based on combined patient–caregiver responses, patients as compared to caregivers, and disease severity (Child–Pugh score). Overall, we found that resources less frequently described as helpful were often identified as needed (i.e., would be helpful) at greater frequency. Moreover, our analysis of resources that would be helpful revealed a notable increase in the frequency of several non-medical domain resources (i.e., support groups, financial, and resource awareness) and mental health services across combined patients–caregivers and specifically among respondents with greater disease severity. This suggests areas for improvement in the medical domain and opportunities for HCP engagement with patients and caregivers to support access to resources in the non-medical domain.

Our findings reflect the literature characterizing social support from family, friends, and other non-family members as a helpful resource [30,31,32]. Respondents in our study caring for patients regardless of disease severity (Child-Pugh score), perceived a lack of support and expressed a need for family members to “step up.” HCPs can involve social workers at various points along the disease to help assess support needs and provide information to both patients and caregivers on local services.

An important finding of this study is the inclusion of the religious belief system/spirituality category as a helpful resource to both patients and caregivers. Our analysis of responses by Child-Pugh score suggests that patients with greater disease severity and their caregivers rely more on their faith and spiritual practices than those with a lower class of disease severity. In a review of the literature, Balboni et al. [33] found that spirituality is important and common among most people with serious illness, and that spiritual community and service attendance is associated with better quality of life measures and mental health. However, spiritual care as a part of medical care is infrequent, and historically HCPs have been reluctant to ask patients about religious and spiritual needs for support and aspects of their well-being [33]. Individualized and respectful approaches to supporting patients’ and caregivers’ spirituality and religious needs could aid in mitigating caregiver burden and improve quality of life and mental health [34]. Positive religious coping and spirituality in other disease conditions has been shown to correlate with improved caregiver burden [35,36]. Because of nurses’ critical role in providing holistic care to patients with ESLD and their caregivers, they are in a unique position to assess patients’ and caregivers’ spirituality and religious needs and refer them to spiritual care professionals and/or religious organizations in the community. However, in clinical practice, nurses and other HCPs prioritize physical care over spiritual care despite the documented importance spirituality and religiosity play in patient and caregiver well-being. This lack of spiritual care may be related to inadequate training, personal beliefs, heavy workloads, and time constraints during patients’ clinic visits [37]. To ensure that spiritual care becomes an integral part of clinical care with the goal of improving patient and caregiver well-being, healthcare organizations need to develop and implement policies that focus on trainings in spirituality, workload distributions, and nurse-to-patient ratio [37,38,39]. As patient advocates, nurses should assume leadership roles in participating in and shaping these policies and nursing protocols to include religious belief system/spirituality assessment and management as an essential dimension of holistic family-centered care [39,40]. The curriculum development in spirituality and religiosity should be initiated and led by nurses. In outpatient clinics, educational modules could be taught on-line and include interactive teaching strategies that cater to individual needs and are created by spiritual care professionals or members of religious organizations in the community.

We identified an unmet need for access to support groups and opportunities to connect with others with shared experiences of liver disease or caregiving, particularly among caregivers of patients with greater disease severity. Participating in online support groups has been shown to have a positive impact on social wellbeing and adapting illness management needs in populations with chronic conditions [41]. Caregivers in our study found online support groups helpful in dealing with the care recipient’s liver disease and wanted opportunities for caregiver centered support groups. Social workers can help patients and caregivers connect to one-line and community-based support groups [42].

The financial status in our study sample indicates that more than half of patients with ESLD (56.7%) and less than half of caregivers (42.1%) listed their financial status as “just enough or cannot make ends meet.” This indicates greater need for financial assistance among respondents, but particularly among patients with increased disease severity (Child class C) and their caregivers (Figure 6). Job loss was often cited as a factor for financial challenges. Adults with CLD are likely to experience financial challenges, which are associated with greater health care utilization and poor health-related quality of life and caregiver burden [8,10,43]. Studies have shown that financial factors can complicate treatment, reduce treatment adherence, and create management challenges [44]. Decreased income can also worsen mental health for caregivers of patients with chronic liver disease [10,45]. To identify patients with CLD and their caregivers who are at a risk for financial distress, Ufere et al. recommend that clinicians routinely begin to screen for patients who are underinsured, unemployed, or have hepatic encephalopathy [10]. Other high-risk patients with ESLD nurses work with in outpatient clinics and could be identified by them include patients who have low socioeconomic status, limited English proficiency, lower health literacy, and lower educational attainment. Although nurses and other HCPs involve social workers in providing practical supports that may help patients and caregivers navigate potential sources of financial burden such as housing, transportation, and health insurance, new models of care are needed [42]. Such models should address the multilevel demographic factors and sociodemographic risk factors that play a role in patient and caregiver financial distress [10]. These models could include social workers, nurses, other health care professionals such as pharmacists, community health workers, and financial advisors [10]. As a collaborative team these professionals would be able to guide patients with ESLD and their caregivers with resources such as health insurance, medical and non-medical expenses, and prescription drug assistance programs [10].

Our findings indicate that both patients and caregivers may benefit from assistance with identifying and connecting to helpful resources. Locating relevant resources can be a burden particularly as the experience of living with and managing liver disease can at times seem overwhelming and unpredictable [2,3,15]. Assessing resource needs and providing patients and caregivers with individualized information early in the disease process, about what to expect when/if the disease worsens, treatments, and symptom management, may help them to cope with the uncertainty of the illness and mitigate potential burdens associated with loss of income due to illness or caregiving responsibilities, symptoms of ESLD and associated complications, and perceived lack of support from other family members.

Within the medical domain, communication cut across the categories HCPs/health care system, liver disease knowledge, and information from HCPs. We identified a desire for improved communication among HCPs as well as more frequent communication with patients and caregivers. Respondents wanted more information about their diagnosis, prognosis, treatment decisions, and what to expect. An exploratory study by Verma et al. [15] identified lack of attention from HCPs and lack of information from HCPs as two salient domains of unmet needs of caregivers of patients being evaluated for liver transplant. Similar health information-seeking behavior has been observed in other diseases as important for self-management [46]. A lack of understanding of their diagnosis, prognosis, and information seeking among patients with ESLD and their caregivers as potential areas for improvement has been documented in the literature [6,7,31,47]. Respondents reported receiving information from HCPs primarily through office visits, which may at times be constrained in time and scope. This suggests an ongoing area for improvement and opportunity for HCPs to better engage patients and caregivers in efforts to manage ESLD. Introducing education about liver disease (e.g., cirrhosis, the patient’s specific diagnosis, potential symptoms, symptom management, etc.) early would help orient caregivers to appropriate resources and materials to prepare them for potential changes in symptom burden and patient- and self-care needs as the course of illness changes [6,47,48,49]. Moreover, learning about liver disease may provide patients and caregivers with a sense of empowerment and a way to cope with and manage caregiving [50].

Patients and caregivers conceptualized medications and procedures as helpful resources to manage symptoms and relieve reported pain associated with ESLD but felt improved access to liver transplantation and medications for pain management would be helpful. Undertreatment of pain and other symptoms in this population is well documented [51,52]. HCPs can support patients’ and caregivers’ efforts to optimize the benefits of medication by providing tailored education and considering early palliative care referral [48,53].

Access to individual counseling for patients and caregivers, but also mental health support during and after hospitalization were identified as specific unmet needs. Ufere et al. [42] found that patients newly diagnosed with ESLD and their caregivers perceived that mental health support would improve their quality of care. Comparison of resources by Child-Pugh score suggests that the mental health services needs of patients and caregivers may be different based on disease severity. Worsening depression and increased caregiver burden has been associated with patient disease severity [54]. Hansen et al. discovered that patients’ with ESLD mental well-being was significantly worse when the quality of their relationship with their caregivers was perceived worse by the caregivers [55]. In one other study, researchers learned that psychological distress was interdependent among decompensated cirrhotic patient–caregiver dyads. Therefore, HCPs should pay attention to how mental health is interrelated within ESLD patient–caregiver dyads and how it affects both members of the dyad [55,56]. HCPs should consider making early referrals to supportive care services (e.g., mental health providers and social workers) to conduct psychosocial assessments, screen for psychological distress, and assist with coordinating referrals including to specialty palliative care [44,57]. Connecting patients and caregivers to stress reduction therapies may also be beneficial [16]. Researchers need to investigate and develop mental health and psychosocial interventions aimed at mitigating effects on mental health, particularly among patients with worse disease severity and their caregivers [14].

There were limitations with this study. The study sample was specific to outpatient hepatology clinics and two organizations in a specific region (the U.S. Pacific Northwest) and may not reflect other settings (e.g., inpatient populations, different health systems), which could influence available resources and reported needs and limits the transferability of the findings. To enhance transferability future studies should include multi-sites. Patient respondents were predominantly male and caregivers were female. Future studies should include different populations of each group to more comprehensively understand potential differences. We reported on survey data that was only collected at a single time-point (baseline) in patients’ and caregivers’ experience of living with ESLD as part of a longitudinal study [17]. Subsequently, we recommend that a longitudinal approach may enhance understanding of how resource utilization may change over time as the disease progresses. The responses obtained from the two open-ended questions provided limited analytical depth. To reach a richer understanding of subjective and dyadic experiences, future research should incorporate in-depth interviews and objective observations. Considering the changes in access to digital health information and telehealth services since this study was conducted, future research should revisit patient and caregiver access to such resources and potential unmet needs in the context of these new technologies. Findings from this study provide a foundation for such future studies to explore the contexts in which resources were perceived as helpful, why specific resources are needed, and potential barriers to accessing resources.

## 5. Conclusions

Comparing patient and caregiver responses by group and disease severity illuminated key differences in helpful resources, unmet resource needs, and emphasized the important role caregivers play in supporting patients with managing liver disease. Proactively engaging both patients and caregivers to identify and access helpful resources early in the course of illness may empower them to manage symptoms and develop informed expectations about living with ESLD. Improving the quality of and access to resources within the medical domain may help to better support patients and caregivers. Involving an interprofessional care approach that includes social workers and referrals to chaplains, support groups, mental health practitioners, and specialty palliative care would facilitate efforts supporting caregiver resource needs and the emotional and mental health needs of both patients and caregivers. However, to fully address the combined multi-dimensional medical and non-medical resource needs experienced by patients with ESLD and their caregivers, new interprofessional models of care are called for. These models should expand beyond the medical model and systematically assess and address needs related to spiritual, emotional, social, and financial dimensions experienced by ESLD patient–caregiver dyads. The development and implementation of such models to provide holistic family-centered care will require committed collaboration among healthcare administrators and HCPs. In particular, nurses working in outpatient clinics should play a major role in the design of new interprofessional models to ensure a holistic approach to family-centered care. To further explore resource utilization and areas for improvement, future researchers should incorporate a longitudinal approach that explores both the individual and dyadic perspectives to understand the specific resource needs at different points in disease progression to test multi-dimensional interventions that facilitate the management of ESLD.

## Figures and Tables

**Table 1:** Characteristics of patient (*N* = 192) and caregiver (*N* = 198) participants.

Characteristic	Patient *n* (%) or M ± SD	Caregiver *n* (%) or M ± SD
Age, years Range	56.5 ± 11.1 23–83	56.8 ± 13.1 19–88
Relationship to caregiver/patient (spouse/partner)	109 (56.8)	116 (58.6)
Live together	143 (74.5)	149 (75.3)
Female (vs. male)	69 (35.9)	150 (75.8)
Education (at least some college)	127 (66.1)	136 (69.4)
Married/partnered	111 (57.8)	137 (69.5)
Hispanic	11 (5.8)	13 (6.7)
White	154 (80.2)	163 (83.6)
Financial status (just enough or can’t make ends meet)	109 (56.7)	82 (42.1)
Employed	25 (13.1)	94 (48.8)
Have health Insurance	187 (98.4)	183 (93.8)
Duration of cirrhosis, years	4.7 ± 5.9	-
Charlson Comorbidity Index Score	3.7 ± 1.9	-
MELD-3.0 score	19.2 ± 5.1	-
Ascites	170 (88.5)	-
Encephalopathy	136 (70.8)	-
Primary etiology		
ETOH	65 (33.9)	-
NASH/Cryptogenic	54 (28.1)	-
Autoimmune/PBC/PSC	25 (13.0)	-
Viral hepatitis	22 (11.5)	-
ETOH & hepatitis C	21 (10.9)	-
Other	5 (2.6)	-
Child–Pugh class ^a^		
Class A	7 (3.7)	-
Class B	89 (46.6)	-
Class C	95 (49.7)	-
Transplant status		
Currently being evaluated	81 (42.2)	-
Evaluated and listed	42 (21.9)	-
Non-candidate: Too sick/comorbidities/obesity/BMI/age, social, support concerns	33 (17.2)	-
Non-candidate: Too early/low MELD/stable/compensated	23 (12.0)	-
Evaluated and deferred listing	7 (3.6)	-
Patient declined/evaluated and declined listing	6 (3.1)	-

Abbreviations: SD, Standard Deviation; ETOH, Ethyl Alcohol; MELD, Model for End-Stage Liver Disease; NASH, Nonalcoholic steatohepatitis; PBC, Primary Biliary Cholangitis; PSC, Primary Sclerosing Cholangitis. ^a^ For one participant, we did not have a Child–Pugh score, as the data was missing.

**Table 2:** Most frequent resources reported in response to open-ended questions 1 and 2 by category within the non-medical domain.

				Exemplary Quotation	
Resource Category	Definition	Question	Frequency ^ab^ *n* (%)	Patient	Caregiver
Social support	Help or assistance received from family members, friends, or others with caregiving activities, practical assistance (e.g., moving), and/or emotional support through socializing and sharing the experience of having ESLD or caregiving.	1	157 (44)	“My niece, who is 6 months younger than myself. She had a liver transplant 3 years ago.”; “Venting to my friends”; “friend with liver disease who received transplant”	“My sister helps me by taking [the patient] to a few appointments.”; “A personal friend who also is coping with liver issues.”; “Meeting people who are going through the same disease.”
		2	45 (15)	“Perhaps communicating with others who have gone through the same thing (Have family & friends who have had liver disease)”; “Meeting more people in my situation at my age”; “Probably being more social”	“Extended family in [state], being more willing and available to help”; “My siblings to help [Patient], too.”; “How a friend can help”; “Someone who could help me when I want to leave for Dr. appointment”
Religious belief system/spirituality	Religious- and spiritual-based resources including belief systems, faith, physical places of worship (e.g., church), and practices (e.g., prayer, reading the Bible).	1	63 (18)	“But I have talked to God - Jesus in guidance in on condition and He sends information my way.”; “daily spiritual reading”; “prayer, surrounding myself with positive environment”	“My faith that God loves [the patient] and holds him in love.”; “Meditation/crystal therapy”; “I read Jesus Calling daily”; “reading the Bible on healing.”
		2	17 (6)	“Being more spiritual & positive minded.”	“Just knowing, The Lord and Praying.”
Support groups	Formal organized groups that meet either in-person or virtually to offer support, such as pre-/post- transplant groups, Al-Anon, diagnosis specific, patient-focused, and caregiver-focused.	1	15 (4)	“Recovery/support groups”; “Facebook PSC groups”	“Counseling from support group”; “Facebook caregivers group.”
		2	43 (14)	“Auto-immune related transplant groups weekly”; “Support groups to help with changes and symptoms.”; “I just learned of a support group and would like to attend”	“Support groups with other family members going through similar situations.”; “Maybe monthly support group; better yet—an online forum”
Financial	Access to health insurance and/or the means to pay for medical or non-medical expenses.	1	6 (2)	“All I have is [insurance name]”; “Good insurance coverage.”	“100% Financial assistance [hospital name]”; “My job and financial situation is secure enough to allow me to help a little and to fly to [state] if needed.”
		2	33 (11)	“$—so don’t have to stress”; “A charity grant or programs to help with the offset in finances (if your [sic] not working).”	“Help w/living expenses while [the patient’s] off work”; “financial help in getting [patient] to appointments”; “Knowing how we can bring in extra money for transplant.”
Resource awareness	Statements that conveyed a lack of knowledge of available resources or uncertainty about resources that would be helpful.	1	5 (1)	“Don’t know any.”	“not sure.”; “don’t have any”
		2	31 (10)	“Unknown—It’s a lot to deal with.”; “so far in stages here I am not sure what else could help”; “not sure what is available.”	“Support groups?? Maybe there are and I don’t know about it yet.”; “just starting out on decomp liver disease journey, looking into more resources.”

^a^ Includes combined patient and caregiver responses (*N* = 360) to question 1 *“What resources have you found most helpful in dealing with your (the care recipient’s) liver disease?”*; ^b^ Includes combined patient and caregiver response (*N* = 307) to question 2 *“What resources would be helpful in dealing with your (the care recipient’s) liver disease?”*.

**Table 3:** Most frequent resources reported in response to open-ended questions 1 and 2 by category within the medical domain.

				Exemplary Quotation	
Resource Category	Definition	Question	Frequency ^ab^ *n* (%)	Patient	Caregiver
Health care professionals (HCPs)/Health care system	The people (e.g., HCPs, clinicians) and medical institutions (e.g., clinics and hospitals) involved in the patient’s care. The perceived quality of medical expertise and care, and quality of communication among HCPs.	1	146 (41)	“Supportive doctors and transplant coordinators.”; “Resources form the [hospital] liver team my Doctors.”	“Great care from the [hospital name] & a medical TEAM that works together—they do a great job.”
		2	33 (11)	“Make sure dr. is well versed liver specialist like mine is”; “go to liver specialist hospital, don’t waste time at smaller facilities.”	“Better connection/sharing info between primary care doctor and [hospital] liver clinic.”; “all doctors involved in [patient’s] care communicating with each other about what is going on (i.e., primary care/gastroenterologist/ER doctors and hospital doctors)”
Liver disease knowledge	Expressed current knowledge of or a desire to learn about liver disease, symptoms associated with liver disease, liver transplantation or other therapeutic treatments, caregiving, and what to expect as the disease progresses. Participants accessed and expressed a need for information from a variety of sources, such as websites, audio recordings, videos, books, magazine articles, prior experiences with caregiving, and caregiving classes.	1	86 (24)	“Medical background personally”; “Study liver disease”; “Reading literature—AA Big Book—liver disease books”	“Past experience-mother passed away from liver disease.”; “Learning from internet about liver function”; “My brother—explains anatomy to me”; “reading all I can about liver disease on the internet and any other sources available”
		2	78 (25)	“Know what kind I have & learning to deal with it”; “Finding out as much as I can regarding liver disease to know as much as possible regarding my disease and how to treat it properly.”; “more information explaining others dealing with my liver disease. Sometimes I feel I’m the only one experiences symptoms, leg cramps, etc.”	“more information about encephalopathy and its effects”; “more information on reactions to medicine; more information on side effects of surgeries; information on progression of the disease”; “More information about what to expect”; “How to best provide help for him”
Information from HCPs	Information patients and caregivers receive from HCPs, clinicians, or social workers involved in the patient’s care that is shared in the form of pamphlets, through the patient portal, and direct communication.	1	38 (11)	“Talking to Dr. for interpretation of signs & blood work”; “we are both learning a lot from good doctors and nurses”; “Input from health providers”	“We (I) have just started dealing with this, so I don’t have much information yet. It has been very helpful talking with the doctor.”; “Literature from doctor for cirrhosis”; “a hand out [the patient] received from [hospital clinic], when [the patient] was first diagnosed”
		2	12 (4)	“Knowing exactly everything that is going to happen—from the Drs.”	“Doctors who are more involved in explaining their course of treatment and why.”; “A social worker to teach about the disease—what to expect, how to protect myself. The doctor is busy diagnosing and can’t cover all the emotional problems that are to be expected.”
Access to medications/procedures	Receiving/access to certain medications, treatment therapies, diagnostic tests, and procedures (e.g., paracentesis, medication, liver transplantation) to help address symptoms, treatment side effects, or provide a level of relief.	1	18 (5)	“Nausea and pain pills with weekly para- and thoracentesis [sic] (very painful).”	“medications”; “body scans”
		2	25 (8)	“More livers, and to have a transplant at a lower MELD score”; “easy access to medicine for managing pain (I can’t take ibuprofen, aspirin or acetaminophen)”	“Giving [the patient] medicine for pain”; “something for his nausea”; “Getting transplant quicker.”
Mental health services	Accessing professional mental health counseling or psychotherapy for either the patient, the caregiver, or for both as a couple.	1	12 (3)	“psychiatry/ counseling”	“Counseling”; “Psychologist, psychiatrist.”
		2	23 (7)	“More frequent counseling”	“Social/Psychological support at [hospital]—think this is lacking now & should be actively provided. [Doctors] & nurses do a great job while [patient] is there but need more while there plus follow-up or resource list while out of hospital.”; “Maybe some 1-on-1 counseling, so each of us can express freely w/o upsetting the other.”

^a^ Includes combined patient and caregiver responses (*N* = 360) to question 1: *“What resources have you found most helpful in dealing with your (the care recipient’s) liver disease?”*; ^b^ Includes combined patient and caregiver response (*N* = 307) to question 2: *“What resources would be helpful in dealing with your (the care recipient’s) liver disease?”*.

## Data Availability

Our data were collected for the research study and are not publicly available to protect study participant confidentiality and due to IRB and HIPAA restrictions. Requests to access the datasets should be directed to the corresponding author.

## Public Involvement Statement

No public involvement in any aspect of this research.

## Guidelines and Standards Statement

This manuscript was drafted against the Consolidated criteria for reporting qualitative research (COREQ) for qualitative research [58].
